# Activation of Mitochondrial Complex II-Dependent Respiration Is Beneficial for α-Synucleinopathies

**DOI:** 10.1007/s12035-015-9399-4

**Published:** 2015-08-29

**Authors:** Christina Fröhlich, Katja Zschiebsch, Victoria Gröger, Kristin Paarmann, Johannes Steffen, Christoph Thurm, Eva-Maria Schropp, Thomas Brüning, Frank Gellerich, Martin Radloff, Rainer Schwabe, Ingolf Lachmann, Markus Krohn, Saleh Ibrahim, Jens Pahnke

**Affiliations:** 1 Department of Pathology (PAT), Translational Neurodegeneration Research and Neuropathology Lab, University of Oslo (UiO) and Oslo University Hospital (OUS), Postboks 4950 Nydalen, 0424 Oslo, Norway; 2 Department of Neurology, Neurodegeneration Research Lab (NRL), University of Magdeburg, Magdeburg, Germany; 3 Department of Neurology, University of Magdeburg/Leibniz Institut for Neurobiology, Magdeburg, Germany; 4 Institute for Mathematical Stochastics, University of Magdeburg, Magdeburg, Germany; 5 AJ Roboscreen GmbH, Leipzig, Germany; 6 LIED, University of Lübeck (UzL), Lübeck, Germany; 7 Department of Bioorganic Chemistry, Leibniz-Institute of Plant Biochemistry (IPB), Halle, Germany; 8 University of Frankfurt, Institute of Clinical Pharmacology/ZAFES, Frankfurt, Germany; 9 Fraunhofer Institute for Cell Therapy and Immunology (IZI), Halle, Germany

**Keywords:** Alpha-synuclein, Parkinson’s disease, Mitochondria, Complex II, Oxidative phosphorylation

## Abstract

Parkinson’s disease and dementia with Lewy bodies are major challenges in research and clinical medicine world-wide and contribute to the most common neurodegenerative disorders. Previously, specific mitochondrial polymorphisms have been found to enhance clearance of amyloid-β from the brain of APP-transgenic mice leading to beneficial clinical outcome. It has been discussed whether specific mitochondrial alterations contribute to disease progression or even prevent toxic peptide deposition, as seen in many neurodegenerative diseases. Here, we investigated α-synuclein-transgenic C57BL/6J mice with the A30P mutation, and a novel A30P C57BL/6J mouse model with three mitochondrial DNA polymorphisms in the ND3, COX3 and mtRNA^Arg^ genes, as found in the inbred NOD/LtJ mouse strain. We were able to detect that the new model has increased mitochondrial complex II-respiration which occurs in parallel to neuronal loss and improved motor performance, although it exhibits higher amounts of high molecular weight species of α-synuclein. High molecular weight aggregates of different peptides are controversially discussed in the light of neurodegeneration. A favourable hypothesis states that high molecular weight species are protective and of minor importance for the pathogenesis of neurodegenerative disorders as compared to the extreme neurotoxic monomers and oligomers. Summarising, our results point to a potentially protective and beneficial effect of specific mitochondrial polymorphisms which cause improved mitochondrial complex II-respiration in α-synucleinopathies, an effect that could be exploited further for pharmaceutical interventions.

**Electronic supplementary material:**

The online version of this article (doi:10.1007/s12035-015-9399-4) contains supplementary material, which is available to authorized users.

## Introduction

Parkinson’s disease is one of the most spread neurological disorders with motor and cognition deficiencies in the world. It is characterised by diverse symptoms, including movement deteriorations, rigidity, bradykinesia, tremor, postural instability and also dementia. Morphological findings in the brain of patients reveal a tremendous loss of dopaminergic neurons in the substantia nigra *pars compacta*. Furthermore, distinct proteinous inclusion bodies (Lewy bodies, Lewy neurites), which are deposited in brain neurons, are the prominent morphological hallmark [1] (reviewed in [2–4]). These inclusions are mainly composed of α-synuclein (aSYN), a 14-kDa peptide localised at presynaptic sites in brain neurons, which is thought to have distinct physiological functions as being implicated in the modulation of synaptic activity, neurotransmitter release and lipid metabolism (reviewed in [5–7]). This peptide plays also a crucial role in pathology of dementia with Lewy bodies and other neurodegenerative diseases like Alzheimer’s disease [8, 9]. Several reports hold the opinion that higher molecular weight aggregates of aSYN are the toxic compound in the brain of Parkinson’s patients, while others denote the monomers and small oligomers to be the crucial peptide species (reviewed in [4, 10]). Numerous mutations in *SNCA* (aSYN gene) causing inherited forms of the disease have been identified, leading to identical clinical outcome, but do appear in only 10 % of all Parkinson’s disease cases [11, 12]. These mutations were often used as basis to generate mouse models of the disease. One very widespread used mutation of aSYN, first discovered in a German family, is the autosomal dominant ‘A30P’ missense mutation, in which alanine at position 30 is replaced by proline leading to enhanced fibril formation [13].

Currently, very intensively debated is the mitochondrial dysfunction, a pathogenic mechanism which has been in focus of research ever since the first description of complex I-deficiency in the substantia nigra, but also in platelets of Parkinson’s disease patients [14–16]. Mutations in the mitochondrial DNA, which are thought to occur during life and especially with increasing age, lead to the production of reactive oxygen species, normally regulated by antioxidants (reviewed in [3]). This process is believed to be disturbed in old age, respectively, in neurodegenerative diseases (reviewed in [17]), and leads to further damage in mitochondria and in pathways for degradation and removal/efflux of aSYN as well as other peptides [18]. Since then, a number of mutations in genes for PTEN-induced putative kinase 1 (*PINK1*), Parkinson protein 2 (*PARK2*), Parkinson protein 7 (*parkk7/DJ-1*), Leucin-rich repeat kinase 1 (*LRRK1*) and others have been shown to induce Parkinson’s disease symptoms, mostly early onset and indistinguishable from sporadic cases [19, 20] (reviewed in [21]). At the same time, it could be revealed that aSYN binds to mitochondria and shifts its fusion and fission balance towards fission [22]. However, the mechanisms leading to the onset of Parkinson’s disease are still unknown and the spatial and temporal sequence of events that trigger pathology remain to be elucidated.

To examine whether the modification of mitochondrial function influences the aSYN pathology of a widely used Parkinson’s disease/dementia with Lewy bodies mouse model, respectively, we generated a novel mouse line as derivative of the A30P mouse line (tg-aSYN-B6) with specific modifications in the mitochondrial genome (tg-aSYN-mtNOD). A related mouse model was previously described in the context of Alzheimer’s disease by Scheffler et al. [23], who showed increased production of ATP in NOD/LtJ-derived mitochondrial conplastic mice with resulting reduced amyloid-β burden and increased microglial function. We compared both mouse models (tg-aSYN-B6 and tg-aSYN-mtNOD) with regard to aSYN load in the brain, motor performance and differences in microglial response as well as number of cortical neurons. Additionally, we determined mitochondrial function of the C57BL/6J background control strains of the generated lines biochemically by examining the respiration rate of isolated brain mitochondria with and without mitochondrial polymorphisms (mtNOD and C57BL/6J). Our findings reveal that the variations in complex II-dependent respiration rates go along with an improvement in behaviour, e.g. motor performance, and the total number of cortical neurons. We could show that it is also accompanied by an increase in the aSYN burden in brains of old transgenic mice, of higher molecular aggregates of aSYN in particular.

Surprisingly, we detected that an increased microglial response with advancing age is lacking contrary to the findings of Scheffler et al. [23] in Alzheimer’s disease mouse models. Furthermore, we found that tg-aSYN-B6 mice lack worsening of motor skills when compared to non-transgenic controls. Thus, we propose to change the denotation of these mice from a Parkinson’s disease mouse model to rather being a general model for cortical α-synucleopathies, e.g. dementia with Lewy bodies.

## Methods

### Mouse Models

C57BL/6J mice (controls) were purchased from Jackson Laboratory (Bar Harbor, ME, USA) and provide the genomic background of the transgenic mouse strains. Tg-aSYN(A30P)-B6 mice were obtained from and described earlier by Kahle et al. [24] and carry the human transgene aSYN with the ‘A30P’ mutation, which is expressed under the neuron-specific Thy1 promoter [24]. To generate the mtDNA conplastic strain tg-aSYN-mtNOD, we used the mouse inbred strain NOD/LtJ, purchased from Jackson Laboratory which has been sequenced previously [25]. The method for generation of a similar mouse strain was already described by Scheffler et al. [23]. Briefly, female mice of the NOD/LtJ strain were mated with male C57BL/6J mice for more than ten generations, always crossing females with C56BL/6J males. Finally, male tg-aSYN-B6 mice were crossed to females of the generated mitochondrial conplastic strain to generate tg-aSYN-mtNOD mice in the genomic C57BL/6J background.

Mice at an age of 50 and 300 days were used for immunohistochemical analyses and ELISA; the behavioural tests were performed using mice starting at 50 days of age until 300 days of age.

For respirometry, we used 100 and 200 days old mice with the specific genomic background, denoted as C57BL/6J and mtNOD mice, and without transgene overexpression. All mice were housed in a climate-controlled environment on a 12-h light/dark cycle with free access to rodent food and water. All experiments were conducted in accordance to the EU and state law of Saxony-Anhalt and approved by the local animal ethics committee.

### Biochemical and Immunohistochemical Analyses

#### Tissue Preparation

Mice were killed by cervical dislocation and transcardially perfused with phosphate-buffered saline (pH 7.4). One brain hemisphere was transferred into 4 % paraformaldehyde for at least 48 h and finally stored in PBS while the second hemisphere was snap-frozen in liquid nitrogen and stored at −80 °C.

#### Isolation of Mitochondria

For functional analyses, mouse brain mitochondria from 100- and 200-day-old mice were isolated as described by Gellerich et al. [26] using a modified protocol according to Clark and Nicklas [27]. For that, one brain hemisphere was transferred into ice-cold MSE-A (225 mM mannitol, 75 mM sucrose, 20 mM MOPS, 1 mM EGTA, 0.5 mM DTT, ddH_2_O, pH 7.4) containing 0.05 % nagarse and homogenised with a glass homogeniser. The homogenates were diluted 1:4 with MSE-A, and centrifuged at 2000*×g* for 4 min. Afterwards, the supernatant was filtered and centrifuged again at 12,000*×g* for 10 min. The pellet containing the mitochondria was re-suspended in 5 ml MSE-A containing 0.02 % digitonin, and after an additional centrifugation step in 20 ml MSE-A at 12,000*×g* for 10 min, the pellet was re-suspended in 100 μl MSE-B (225 mM mannitol, 75 mM sucrose, 20 mM MOPS, 0.1 mM EGTA, 0.5 mM DTT, ddH_2_O, pH 7.4). Protein concentration was determined using the Bicinchoninic Acid Kit (Sigma-Aldrich Chemie GmbH, Steinheim, Germany) and the SunriseTM Reader (Tecan Deutschland GmbH, Crailsheim, Germany).

#### Respirometry

**Fig. 1:**
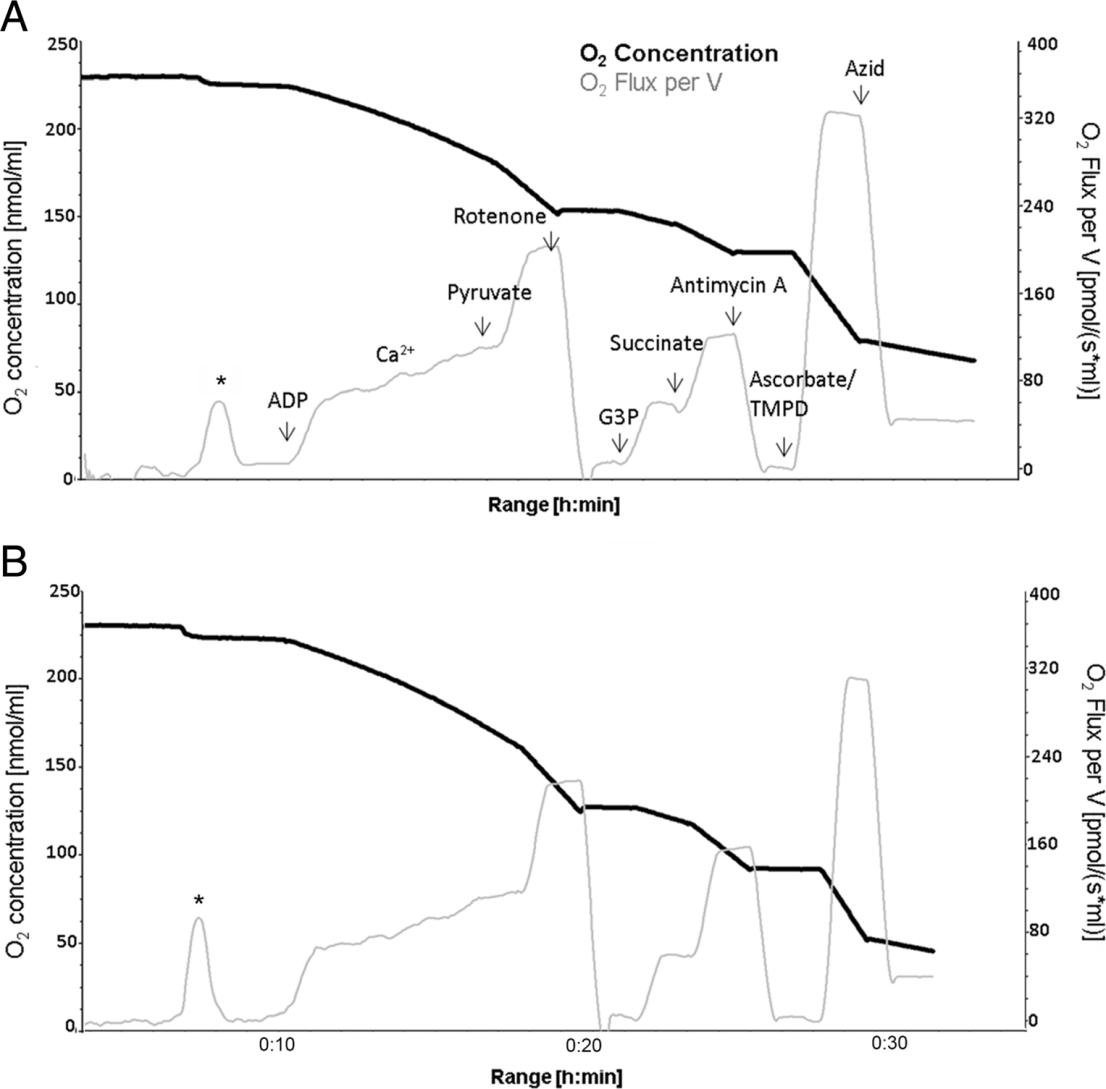
Representative respirograms of 200-day-old C57BL/6J (**a**) and mtNOD mice (**b**). Shown is the sequential addition of substrate according to the first experimental approach ‘protocol I’ (see Table 1 in Supplementary Materials) and the resulting changes in O_2_ concentration (*black line*) and O_2_ flux (*grey line*), which were further evaluated. The addition of mitochondria to the medium results in an immediate increase in oxygen consumption (first peak in A and B marked with *asterisk*) leading to equal oxygen concentration between mitochondria suspension and medium. *G3P*—glycerol-3-phosphate

Respirometry was performed using OROBOROS oxygraph-2k (Oroboros Instruments, Innsbruck, Austria) equipped with Clark-type oxygen electrodes. Respiration of mitochondria (0.06 mg protein/ml) was measured at 30 °C in a medium (BIM-1000) containing 120 mM mannitol, 40 mM MOPS, 5 mM KH_2_PO_4_, 60 mM KCl, 5 mM MgCl_2_ and 1 mM EGTA, pH 7.4. Fluorimetrically measured Ca^2+^ concentration in this medium (Ca^2+^_cyt_) was 11 nM. To study the substrate specific as well as the respiratory chain complex-dependent rates of state 3 (maximum) respiration, four different protocols were used (see Supplementary Materials Table 1). As shown in Fig. 1 and in Supplementary Material, protocols I (and II) were started under conditions of very low Ca^2+^ (11 nM). For that, mitochondria were incubated in a 1 mM EGTA-containing BIM-1000 medium. In the presence of 10 mM glutamate and 2 mM malate (protocol I), the addition of 2 mM ADP adjusted the maximum possible respiration with these substrates (state 3_glu/mal_), which was stimulated by two subsequent Ca^2+^ additions. This procedure allowed testing the activation of mitochondrial substrate supply by extra-mitochondrial Ca^2+^ [28]. After addition of 10 mM pyruvate, the complex I-dependent state 3_com.I_ was reached. Afterwards, 1.5 μM rotenone, an inhibitor of complex I fully repressed the complex I-dependent respiration. The following addition of 10 mM glycerol-3-phosphate adjusted the state 3_G3P_, whereas the state 3_suc_ followed succinate addition (complex II). Now antimycin A, an inhibitor of complex III, fully withdrew the oxidation of these both substrates. Finally, ascorbate/TMPD allowed measuring the maximum oxidation rate of reduced cytochrome c by cytochrome c oxidase. To correct this rate by unspecific oxygen consumption, azide (inhibitor of cytochrome c oxidase) was applied. In protocol II, 10 mM α-ketoglutarate was used in the second incubation step, instead of 10 mM glutamate. Protocols III and IV were performed in the absence of EGTA allowing a larger endogenous Ca^2+^ content, sufficient to fully activate the Ca^2+^ stimulated substrate supply. Starting with pyruvate/malate, at first a small amount of ADP was added to adjust a partially activated state of respiration. After a short time, the rate of respiration decreased again (state 4). Then, 2 mM ADP was added to reach the state 3_pyr/mal_. In the next step, addition of 10 mM glutamate led to state 3_com.I_ and the further addition of succinate adjusted the state 3_com.III_. To check a possible limitation of the oxidative phosphorylation (OXPHOS) by a dysfunctional ATPase, we performed a stepwise FCCP addition causing an increasing uncoupling of OXPHOS. Protocol IV started with adding 10 mM glutamate and 2 mM malate and reaching state 4_glu/mal_ and state 3_glu/mal_, before 10 mM pyruvate then adjusted state 3_com.I_. After the application of 10 mM succinate, state 3_com.III_ was measured. This is due to the fact that the sum of state 3_com I_ plus state 3_com II_ is generally higher that state 3_com III_. Therefore, the complex III is rate limiting under these conditions. Rotenone inhibited the complex I-dependent respiration allowing the registration of state 3_suc_. Finally, 125 μM atractyloside allowed the measurement of state 4_atractyloside_. These protocols enabled the examination of maximum oxidation rates of all used substrates and the maximum flux rates of respiratory chain complexes I, II, III, IV and V. Moreover, several ratios and special properties, e.g. respiratory control ratio (RCR) and non-phosphorylating (state 4) respiration, were analysed. The oxygen concentration in air-saturated medium was considered to be 200 nmol O_2_/ml at 95 kPa. Mitochondrial protein-related oxygen consumption was calculated from the time derivative of oxygen concentration (Datagraph Analysis software, Oroboros Instruments, Austria). Data are given in nanomole O_2_/minute/milligram mitochondrial protein.

#### Enzyme-Linked Immunosorbent Assays

For quantitative analysis, we used ELISA kits (AJ Roboscreen GmbH, Leipzig, Germany) to detect monomeric but also higher molecular forms of aSYN (MONO kit). Therefore, snap-frozen mice brain were gently thawed at 4 °C and homogenised (SpeedMILL PLUS, Analytik Jena AG, Germany) once for 30 s before transferred into 20 volumes (*w*/*v*) carbonate buffer (100 mM Na_2_CO_3_, 50 mM NaCl, ddH_2_O, pH 11.5) and homogenised again for 20 s. After centrifugation at 18,500*×g* for 20 min, the supernatant was captured and denoted carbonate fraction. The remaining pellet of the carbonate fraction was used to produce the guanidine fraction by adding 8 volumes (*w*/*v*) 5 M guanidine hydrochloride buffer (5 M Guanidin/HCl, 50 mM Tris/HCl, ddH_2_O, pH 8) to the initial tissue weight and mixing gently at room temperature for 3 h before centrifuging again. The supernatant was collected as guanidine fraction. The ELISA was performed according to the manufacturer’s instructions. The bound HRP-coupled antibody complex was visualised by TMB/H_2_O_2_ staining and absorption measured using a PARADIGM spectrophotometer (Beckman-Coulter/Molecular Devices, Germany). Protein content was determined by spectrophotometric measurements (ScanDrop, Analytic Jena AG, Germany).

#### Immunohistochemistry

For immunohistochemistry, paraformaldehyde-fixed brain hemispheres were dehydrated, paraffin-embedded and cut into 4 μm-thick coronal sections as previously published [23, 29–31]. After de-paraffinisation and re-hydration, immunostaining was performed using the BOND-MAX Autostainer (Leica Microsystems GmbH/Menarini, Germany). Therefore, endogenous peroxidase was blocked (5 min) and epitopes retrieved for 5 min with citrate (for anti-human aSYN (clone 5G4, AJ Roboscreen), anti-neuronal nuclei (NeuN, Chemicon/Millipore), EDTA buffer pH 8.5 (for Iba1, WAKO) or enzymatic (for glial fibrillary acidic protein [GFAP], DAKO). Antibodies were incubated for 30 min with following dilutions: aSYN 5G4 (1:6000), NeuN (1:500), Iba1 (1:1000) and GFAP (1:500), and detected with BOND-MAX Bond Polymer Refine Detection kit (DAB R30). Additionally, sections were counterstained with haematoxylin. Slides were digitalised using a Mirax/Pannoramic automated slide scanner at a resolution of 230 nm [32]. Further analyses were performed using the Pannoramic viewer software (3DHISTECH Ltd., Hungary) and the BZ Analyser software (Keyence, Germany).

#### Semi-quantitative Analysis of Immunohistochemistry

To examine differences in microglia response and distribution as well as quantity of neurons, the partial area covered by Iba1-positive cells and the number of NeuN-positive cells in the cortex, respectively, of all mouse strains were determined by using the Hybrid cell count function of the BZ Analyser (Keyence, Germany). Therefore, four pictures were taken from the cortex of each mouse using the Pannoramic viewer (3DHISTECH Ltd., Hungary) and the ratio of stained cells area per cortex area was analysed. The mean value was calculated of at least five mice per mouse strain for both points in time, 50 and 300 days, respectively.

### Behavioural Testing

#### Rotarod

Locomotor activity of transgenic and control mice was characterised by the Rotarod performance test. For training, mice had to walk on a rotating rod (Ugo Basile, Italy) with a constant speed of 4 rpm for 1 min. After a recovery time of at least 30 min, they had to perform three trials on an accelerating (4–32 rpm) rod with a maximum duration of 4 min and an inter-trial interval of 30 min. The time in which mice fell of the rod was documented together with the time they first performed passive rotations (without actively walking, but gripping the rod), 240 s were set as cut-off time. Performance of mice that did not fell of the rod was censored at 240 s; the proportion of censoring in each mouse model was also determined. Mice started from an age of 50 days and had to perform the test every 4 weeks until reaching the age of 300 days. At least five mice per mouse strain and time point were analysed.

#### Pole Test

To evaluate movement deteriorations and diminished orientation as characteristics of PD, the pole test was performed. Therefore, a wooden pole (50 cm height, 0.8 cm diameter) with a rough surface was placed in the home cage. Cage mates were removed during this time. The mouse was placed head upwards near the top of the pole and the time till it turns head downwards was documented as well as the time it reaches the floor with all four limbs. Mice were trained three times with a recovery time of at least 10 min at the first day. At day 2, five trials were evaluated of which the best trial was used for evaluation. Data of mice slipped or fallen of the pole were not taken into account. Mice started from an age of 50 days and had to perform the test every 4 weeks until reaching the age of 300 days. At least five mice per strain and time point were analysed.

#### Statistics

Statistical analyses of the ELISA, immunohistochemistry and respirometry data were performed using GraphPad™ prism 6 and one-way ANOVA followed by Sidak’s multiple comparison test, or Kruskal-Wallis test followed by Dunn’s multiple comparison test if normal distribution was not given, respectively. Significance was reached when *p* ≤ 0.05.

Statistical analyses of behavioural tests were performed by the Institute for Mathematical Stochastics using JMP 9 (SAS institute, Heidelberg, Germany). Here, we used one-way ANOVA followed by Bonferroni’s multiple comparison test for the evaluation of the rate of censoring in the Rotarod data set. As variance stabilising transformation of data (arcsine-square root transformation) did not change the outcome of statistical analysis in this case, we spared and used the raw data instead. Significance was reached when *p* < 0.05. The Q-Q plot of the Rotarod data shows that these are distributed normally until reaching the censoring cut-off time point. Additional analyses were performed using R 3.0.2 (R Development Core Team, www.r-project.org) and the package ‘lmec’. This function fits ‘Linear Mixed-Effects Models with Censored Responses’ as is provided by this data set. Significance was reached when *p* < 0.05.

Due to a better adjustment to normal distribution, the ‘pole test’ data set was non-parametrically transformed to mean ranks and fitted to a linear mixed model with fixed effects for genotype and random effects for animals. The analyses were performed using the REML (restricted maximum likelihood) approach and additional two-sided *t* test with adjusted degrees of freedom to determine differences only for the data of main interest, collected between 25 and 33 weeks of age and additional between 37 and 41 weeks of age. Here again, significance was reached when *p* < 0.05 (for further information, also refer to the methods text in the Supplementary Material).

## Results

### Respirometry of C57BL/6J and mtNOD Mice

To analyse functional consequences of the mitochondrial polymorphisms, we performed respirometric investigations of functional properties in isolated mitochondria. Oxygen consumption was measured using four different multiple substrate protocols (see methods, refer to Supplementary Material Table 1) to investigate the function of various complexes of the electron transport chain (ETC) in non-transgenic mice with mtNOD alterations and compared them with C57BL/6J controls.

**Fig. 2:**
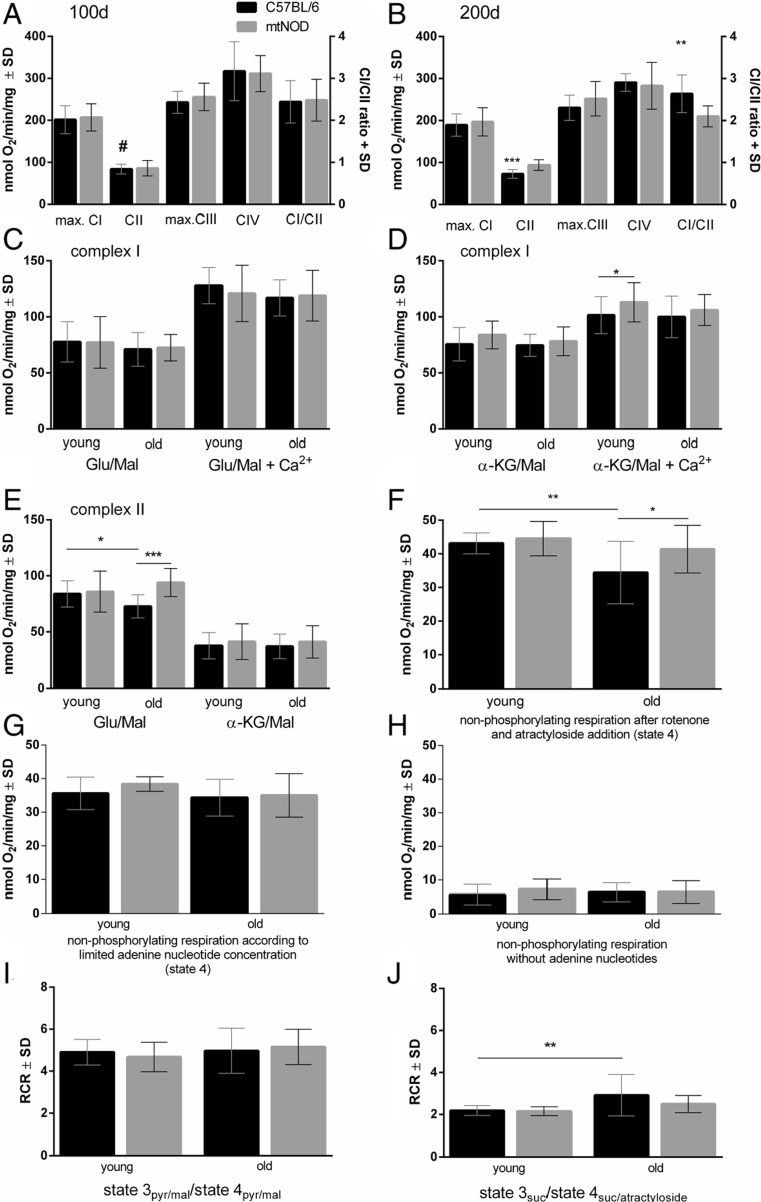
Substrate-specific and respiratory-chain-complex-limited rates as well as non-phosphorylating rates of respiration of brain mitochondria isolated from control (C57BL/6J) and mtNOD mice brains. Rates of respiration as well as ratios were measured as described in ‘Methods’. The complex II-dependent rates of state 3 respiration in the presence of glutamate/malate were significantly increased in 200-day-old mtNOD mice as compared to controls (**b**; **e** for detail), and also to young (100 days) controls (**a**, *number sign* indicates significant difference to 200-day-old mitochondria in **b**). In the presence of α-ketoglutarate (KG)/malate, the rates of complex II-dependent respiration are clearly lower as compared to those with glutamate and are not affected by mtNOD polymorphisms or age (**e**). No differences are detectable in other complexes of the electron transport chain (**a**, **b**). The rates of state 3_glu/mal_ coefficient are stimulated by Ca^2+^ to about 163 % without age- or polymorphism-related changes (**c**), whereas the state 3_KG/mal_ coefficient (**d**) increased only about 34 %. Nonetheless, the mtNOD polymorphisms enhanced the Ca^2+^ activated state 3_KG/mal_ coefficient in 100-day-old animals significantly by 11 % indicating an increased activity of α-ketoglutarate dehydrogenase [28]. After Ca^2+^-addition to the system, cytosolic Ca^2+^ activates the mitochondrial glutamate uptake via aralar, the glutamate aspartate carrier as part of the malate aspartate shuttle. This mechanism is responsible for pyruvate supply to mitochondria. In contrast, Ca^2+^ can after its uptake by mitochondria modulate the activity intramitochondrial dehydrogenases [57]. Different modes of non-phosphorylating respiration were determined for complex I- and complex II-dependent substrates. After inhibition of the adenine nucleotide carrier with atractyloside, the non-phosphorylating respiration with succinate as substrate was smaller in old controls (C57BL/6J) compared old mtNOD mice and to young control mice (**f**). The state 4_glu/mal/pyr_ respiration, in the presence of 75 nM adenine nucleotides (mainly ADP), showed no changes due to age and polymorphism (**g**). The rates of non-phosphorylating respiration without added adenine nucleotides revealed no changes caused by age and polymorphism (**h**). Additionally, respiratory control ratios (RCR) were calculated for complex I-dependent respiration (**i**) and complex II-dependent respiration (**j**). No changes are observable in complex I-dependent respiration, whereas old control mitochondria show significantly greater RCR_suc/atractyloside_ values as compared to young controls in complex II-dependent respiration (**j**). Data is presented as means ± SD (*n* ≥ 11), *, #*p* ≤ 0.05; **p ≤ 0.01; ***p < 0.001 (one-way ANOVA followed by Sidak’s multiple comparison test and Kruskal-Wallis test followed by Dunn’s multiple comparison test, respectively)

Mean of state- and complex-specific rates of respiration of at least six experiments for young and old mice are shown in Fig. 2a, b. In young animals, no significant difference could be observed between mtNOD and C57BL/6J, respectively, for complexes I, III and IV related rates of state-specific respiration (refer to Supplementary Material Table 2). However, in old C57BL/6J animals (200 days) the state 3_suc_ respiration (complex II) was significantly *decreased* as compared to young C57BL/6J mice (100 days), and is also significantly lower as compared to age-matched old mtNOD mice (Fig. 2a, b). Since the complex I-dependent rates were not changed between the different strains, the ratio of complex I/complex II was higher in old C57BL/6J mice as compared to mtNOD mice as well (Fig. 2a, b). Ratio changes are usually a sensitive indicator for complex I-dependent impairments if complex II-respiration is not changed [33]. The substrate-specific rates of complex I-dependent state 3 rates are shown in Fig. 2c–e. The results were obtained either by starting the measurements with glutamate (protocol I) or α-ketoglutarate (protocol II). No significant changes can be observed in the respiratory rates of state 3_glu/mal_ (complex I) without added Ca^2+^. The extent of the Ca^2+^ activation of state 3_glu/mal_ (approximately 163 %) was larger than that of state 3_KG/mal_ (approximately 134 %) as it is known from earlier measurements [26], but was not changed due to age or mitochondrial polymorphism (refer to Supplementary Material Table 2 for a summary of the different metabolic states). Whereas, the absolute rates of state 3_KG/mal+Ca_^2+^ were elevated in young mtNOD animals (Fig. 2d).

Succinate oxidation rates (Fig. 2e) in the presence of glutamate (complex II) were significantly diminished in mitochondria of 200-day-old C57BL/6J mice, as described above.

Interestingly, the rates state 3_suc_ respiration measured with protocol II in the presence of α-ketoglutarate was generally smaller than measured with protocol I in the presence of glutamate probably as a result of different levels of the inhibition by oxaloacetate, as discussed later.

The different protocols allowed us to measure three different resting states. The rates of non-phosphorylating respiration without addition of adenine nucleotides (protocol III; Fig. 2h) are lower than the rates measured after addition of low amounts of ADP (state 4, protocol III; Fig. 2g). This is achieved since the latter rates are partially caused by phosphorylation of ADP supplied by extramitochondrial ATPases that are present in isolated mitochondria (Fig. 2g, h). Only complex I-dependent substrates contribute to these rates of respiration, which exhibited no differences caused by mitochondrial polymorphisms and/or ageing. The third kind of resting state was measured with protocol IV after rotenone and atractyloside addition (Fig. 2f). These rates of respiration are caused by succinate dehydrogenase (SDH) only. We found the non-phosphorylating respiration, after rotenone and atractyloside addition (state 4_suc/atractyloside_), in 200-day-old C57BL/6J mitochondria to be decreased when compared to mitochondria of young C57BL/6J mice (100 days) and to old mtNOD mice (Fig. 2f). This result reflects similar differences as measured under state 3 conditions (Fig. 2a, b) and is probably caused by reduced SDH activity. The quality of coupling between respiratory chain and phosphorylation is described by the respiratory control ratio (RCR). The RCR values for complex I, calculated for the respective state 3 and resting states, are not changed by age or strain (Fig. 2i). Interestingly, RCR, calculated from complex II state 3 and state 4 after atractyloside addition, differs significantly between 100-and 200-day-old C57BL/6J mitochondria (Fig. 2j), confirming former results. The increasing RCR (state 3_suc_/state 4_suc/atractyloside_) is mainly caused by the decreased state 4_suc/atractyloside_ respiration. In contrast, there is no significant difference between the RCR of old C57BL/6 and mtNOD mice but the rates of state 3_suc_ (+20 %) and state 4_suc/cat_ (+16 %) are significantly higher in mtNOD compared to C57BL/6 mice.

### Biochemical and Immunohistochemical Analyses

#### Immunohistochemistry

**Fig. 3:**
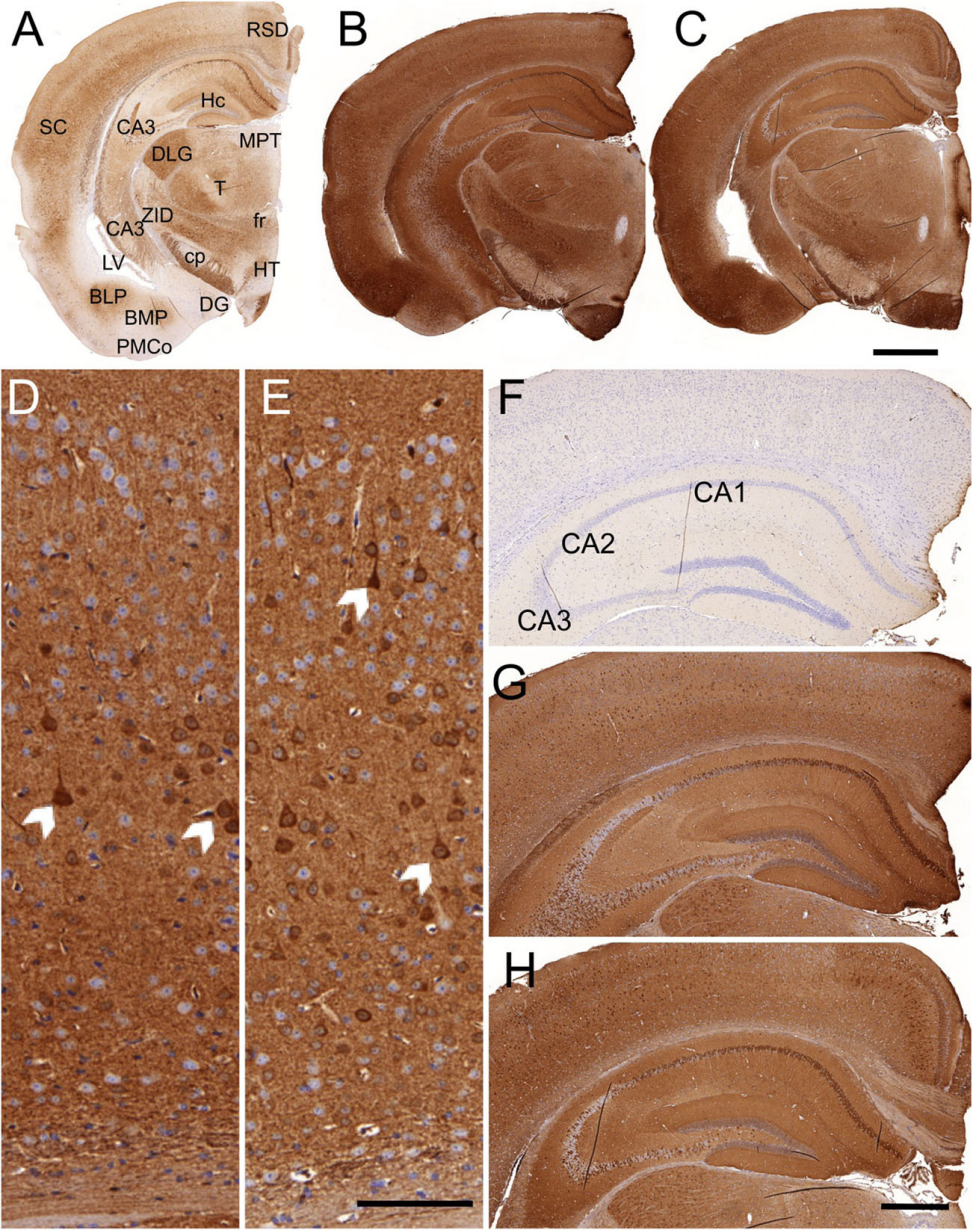
aSYN-immunoreactivity of old tg-aSYN-B6 (**b**) and old tg-aSYN-mtNOD mice (**c**). Sections were immunostained using an anti-human-aSYN antibody, clone 5G4, and were counterstained for nuclei with haematoxylin. Localisation of anatomical structure is shown in **a** (reduced staining intensity). Intense immunolabelling can be detected in the neuropil of the cortex (*SC*), hippocampus (*Hc*), thalamus (*T*), basolateral amygdala (*BLP*), geniculate nucleus (*DLG*) and zona incerta (*ZID*), whereas staining of hypothalamus (*HT*), mediale amygdala (*BMP*, *PMCo*) and dentate gyrus (*DG*) is less intense. aSYN load in the cortex and hippocampus of 300-day-old tg-aSYN-B6 and tg-aSYN-mtNOD mice is shown in **d**–**h**. The magnified cortex regions (**d**, **e**) reveal a slight difference between old tg-aSYN-B6 (**d**) and tg-aSYN-mtNOD mice (**e**), with tg-aSYN-B6 mice stained more intense. *Arrows* point to intensely stained neuronal somata. The most vigorous staining can be observed in cortical layer V (**d**,**e**). In comparison to tg-aSYN-B6 (**g**) and tg-aSYN-mtNOD mice (**h**), no staining is detectable in the hippocampus of controls (**f**). The pyramidal cells show the most intense staining in the CA1 and CA3 regions whereas the CA2 region sparse any labelling and is aSYN-negative (**g**, **h**). *RSD* retrosplenial dysgranular cortex, *Hc* hippocampus, *MPT* medial pretectal nucleus, *SC* somatosensory cortex, *CA* cornu ammonis, *DLG* dorsal lateral geniculate nucleus, *T* thalamus, *ZID* zona incerta, dorsal part, *fr* fasciculus retroflexus, *cp* cerebral peduncle, *LV* lateral ventricle, *BLP* basolateral amygdaloid nucleus, posterior part, *BMP* basomedial amygdaloid nucleus, posterior part, *DG* dentate gyrus, *HT* hypothalamus, *PMCo* posteromedial cortical amygdaloid area; *Scale bar* = 1000 μm in **a**–**c**; 100 μm in **d**, **e**; 500 μm in **f**–**h**

Immunostaining of coronal paraffin-embedded brain sections of both transgenic mouse models with anti-human aSYN clone 5G4 revealed an intensive signal in various anatomical regions of the brain (Fig. 3). In detail, the cortex, thalamus, hippocampus, basolateral amygdala, geniculate nucleus and zona incerta were stained most intensive, while the hypothalamus, medial amygdala, dentate gyrus and other structures only show less intense immunolabelling.

The comparison of young and old mice of both strains, tg-aSYN-B6 and tg-aSYN-mtNOD, showed no differences in intensity of the signal (not shown). Here, neither an increase between 50- and 300-day-old tg-aSYN mice, respectively mice with mitochondrial DNA polymorphism was found, nor between both models (see Fig. 3). The enlarged cortex sections in Fig. 3d, e reveal a slightly more intense staining of 300-day-old tg-aSYN-B6 compared to tg-aSYN-mtNOD mice. At this point, the most vigorous labelling can be observed in the neurons of cortical layer V.

Examination of the hippocampus revealed no obvious variances between both mouse models but points out that distinct regions of the hippocampus show different immunoreactivities, i.e. missing aSYN expression in the pyramidal cells of the CA2 region in contrast to a moderate expression pattern in the CA1 and CA3 regions (see Fig. 3g, h).

**Fig. 4:**
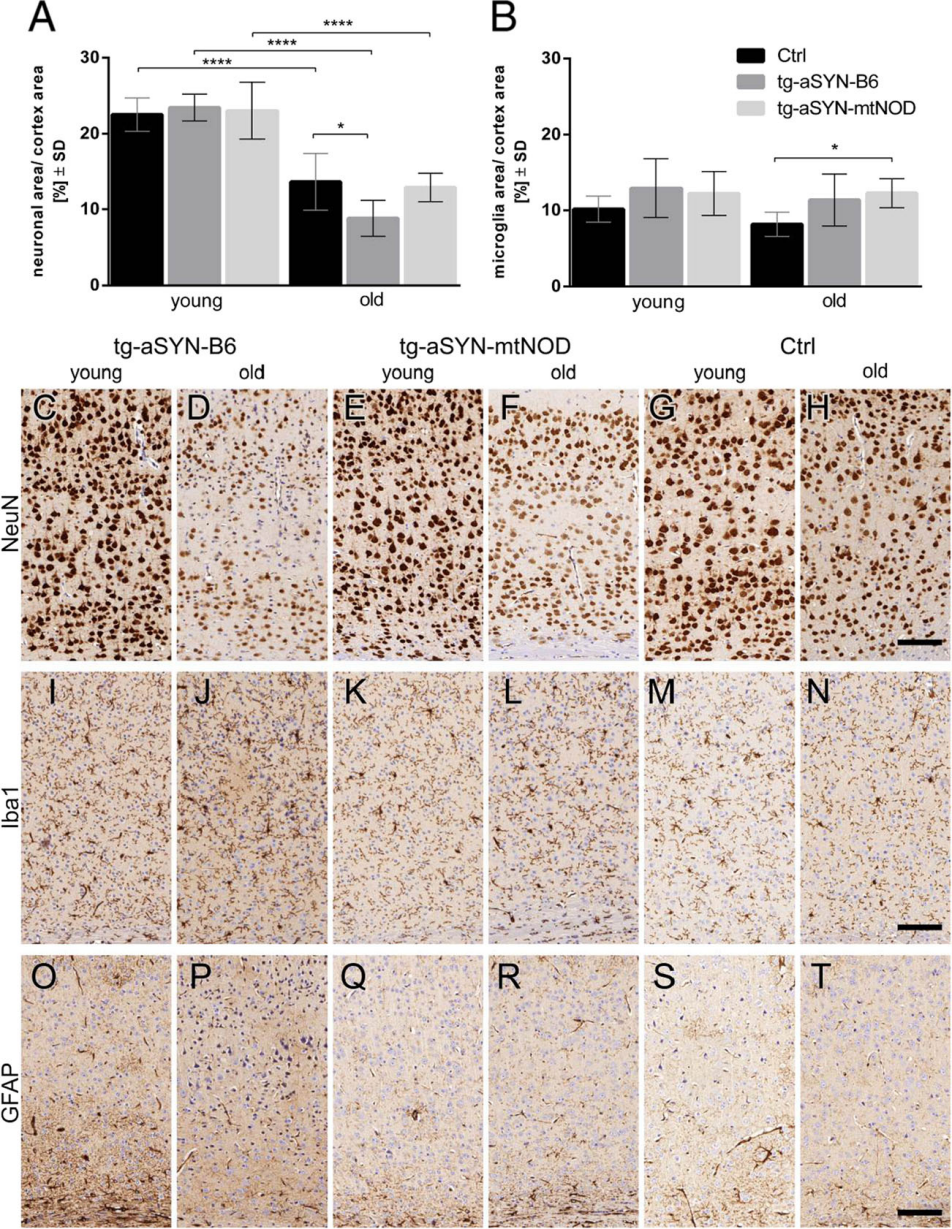
Immunohistochemical analyses of young and old tg-aSYN-B6, tg-aSYN-mtNOD and C56BL/6 control mice (Ctrl). Semi-quantitative analysis of immunolabelling reveals significant differences in neuron integrity between young and old mice and especially between old tg-aSYN-B6 and old control mice (**a**). Examination of microglial response shows a significant difference between 300-day-old tg-aSYN-mtNOD and 300-day-old control mice (**b**). Young tg-aSYN-B6 (**c**) and tg-aSYN-mtNOD (**e**) mice show a more intensive NeuN-staining as compared with old mice (**d** and **g**); old tg-aSYN-B6 mice also reveal less neurons in cortical layer V. There are hardly any changes visible in the GFAP labelling (**o–t**), whereas Iba1 labelling shows slightly stronger microglial activity in old transgenic mice (**j**, **l**) compared with C57BL/6J control mice (**n**). *Scale bar* = 100 μm in **c–t**. Data is presented as means ± SD (*n* ≥ 5), *p ≤ 0.05; **p ≤ 0.01; ***p < 0.001 (one-way ANOVA followed by Sidak’s multiple comparison test)

To investigate whether immune response and neuronal integrity are altered in transgenic mice compared with controls, brain sections were immunostained with specific antibodies. Figure 4 displays the immunohistochemical analyses of NeuN, Iba1 and GFAP labelling and a comparison between young and old tg-aSYN-B6, tg-aSYN-mtNOD and control mice, respectively. Here, a reduced intensity of NeuN immunoreactivity is detectable in old mice (C-H). Iba1 staining shows only slight visually detectable differences between age and strains (I-N), whereas GFAP immunostaining sparse any visible differences and as it is hard to quantify (O-T).

#### Semi-quantitative Analysis of Immunohistochemistry

For standardised detection of differences in area covered by neurons and microglial response, we performed semi-quantitative evaluations. Determination of NeuN-positive area (neurons) in the cortex of transgenic and control mice shows no differences between young mice, but revealed significantly larger neuronal area in the cortex of old C57BL/6J mice as compared to tg-aSYN-B6 mice. Mice with mtNOD polymorphisms show fully rescued neuronal integrity, as the neuronal area revealed no differences compared to control mice. As expected, all mouse strains display a significant decline in neuronal area corresponding to age (Fig. 4a). In contrast, no differences in microglial response were detected in the cortex of young mice, and between young and old animals of any strain. Interestingly, tg-aSYN-mtNOD mice show a significantly and tg-aSYN-B6 mice a tendentially increased microglial response compared to C57BL/6J mice (Fig. 4b).

#### ELISA

**Fig. 5:**
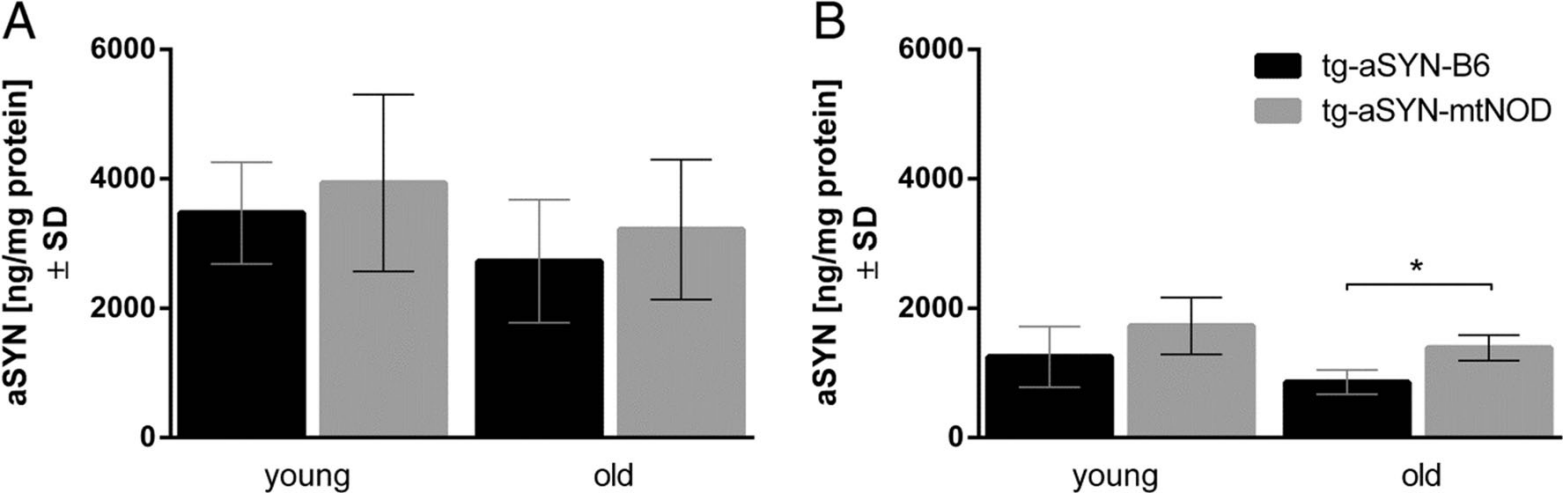
Quantification of aSYN in the brains of tg-aSYN-B6 and tg-aSYN-mtNOD mice. The carbonate-soluble aSYN levels show no differences in young and old mice and between the mouse models (**a**), whereas mice with mitochondrial polymorphism show significantly higher amounts of guanidine-soluble aSYN at 300 days of age when compared to tg-aSYN-B6 mice (**b**). Note the unchanged amount of total aSYN between both time points. Data is presented as means ± SD (*n* ≥ 6), *p ≤ 0.05 (one-way ANOVA followed by Sidak’s multiple comparison test)

To further evaluate observations made using immunohistochemical analyses, protein was isolated from the brain of mice and ELISA measurements were performed. Surprisingly, we found the tg-aSYN-B6 mice to have significantly less guanidine-soluble aSYN (aggregates and higher molecular species) in 300-day-old mice as compared to tg-aSYN-mtNOD mice. Additionally, the same tendency was visible for 50-day-old mice (Fig. 5b). In contrast, the carbonate-soluble aSYN concentration (monomers) showed no differences between ages and strains (Fig. 5a). The latter may verify that the production of aSYN(A30P) is driven by the same promoter activity in both transgenic mouse models.

### Behavioural Testing

#### Rotarod

To determine whether the mitochondrial DNA polymorphisms of NOD/J mice have an impact on the motor performance of transgenic mice, we performed the Rotarod performance test. At least five male mice were analysed in each group, i.e. tg-aSYN-B6, tg-aSYN-mtNOD and C57BL/6J (control) mice. According to the cut-off time of 240 s, first analyses were conducted with respect to the percentage of censoring. The results reveal a significantly higher amount of censored data in case of tg-aSYN-mtNOD mice (75 %) as compared to tg-aSYN-B6 mice (34 %) and control mice (23 %), calculated for the points in time in which the greatest differences were expected, i.e. 25–33 weeks (not shown). Due to the large portion of censoring (mice reaching 240 s), further statistical analyses required a better-fitted model.

To verify the influence of the mitochondrial polymorphisms on the rotarod performance during the defined periods (9–41 weeks), a linear statistical model was adjusted to the obtained data set. This model was used to compare tg-aSYN-B6 and tg-aSYN-mtNOD mice, and tg-aSYN-B6 and ctrl mice, respectively.

**Fig. 6:**
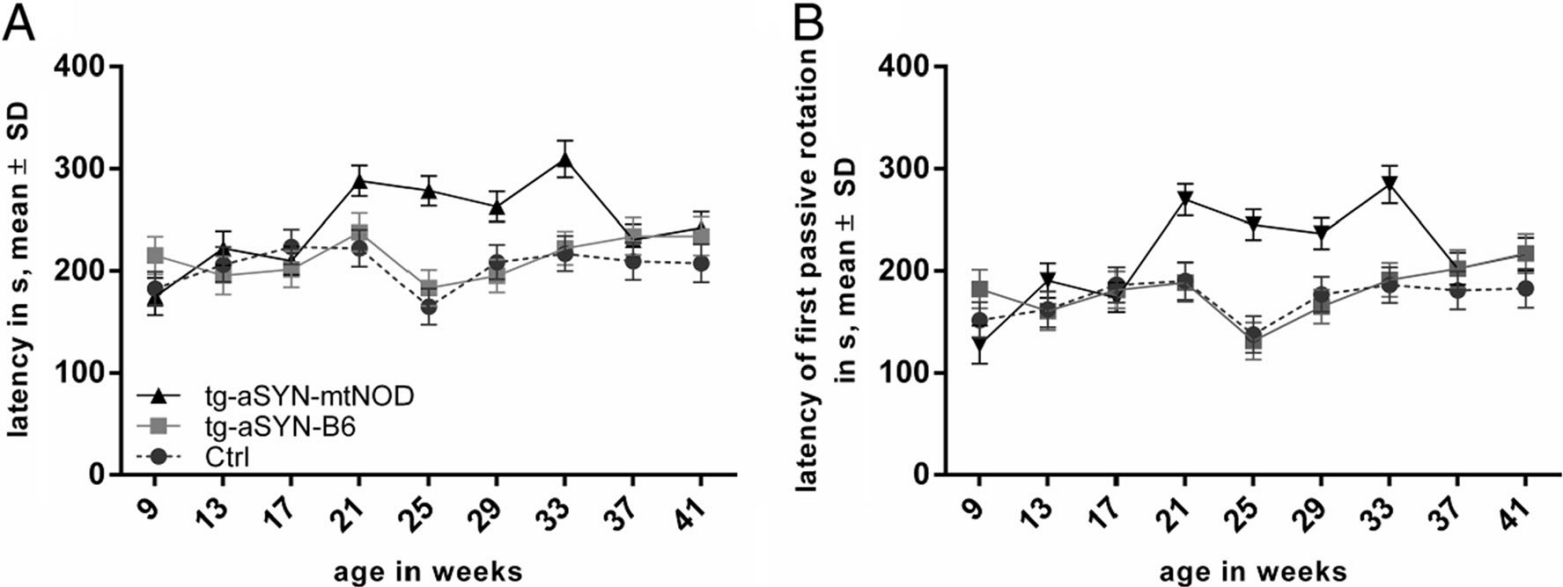
Age-dependent alteration in motor behaviour of tg-aSYN-B6, tg-aSYN-mtNOD and control mice (C57BL/6J). Latency of rotarod performances (**a**) and time until first passive rotation (**b**) of tg-aSYN-B6 mice and controls show no differences until the age of 41 weeks (end date), whereas tg-aSYN-B6 and tg-aSYN-mtNOD mice differ significantly along the time points, with the most significant differences between 25 and 33 weeks of age. The evaluation of censored data reveals a significantly greater amount of censoring (reaching the cut-off time of 240 s) in case of tg-aSYN-mtNOD mice as compared to tg-aSYN-B6 mice and control mice (not shown). Data is presented as means ± SD (*n* ≥ 5, male) (LMEC, for further information, see text in Supplementary Material)

Given the fact that each mouse was measured at several time points, it was recommended to fit these repeated measurements using precisely a ‘linear mixed model with random effects’. However, the great proportion of censoring in each mouse model led to requirement of an additional adjustment to this statistic model. Performing Linear Mixed-Effects Models with Censored Responses (LMEC), we were able to statistically verify that tg-aSYN-mtNOD mice perform significantly better as compared to tg-aSYN-B6 mice in both defined parameters, i.e. maximum latency on the rod and time until first passive rotation (*p* = 0.0158, *p* = 0.0318) in the examined time period. However, highest differences (*p* < 0.0002) were found at mid-age time points between 25 and 33 weeks of age for both, time until first passive rotation and maximum latency on the rod (Fig. 6). No changes in motor performances were found between tg-aSYN-B6 and control mice, indicating missing phenotypic alterations in the transgene mouse models (Fig. 6). The linear influence of age was calculated for the rotarod performance to control for age-related effects in the mouse models.

As an effect of training was statistically excluded, it was most interesting to detect that age has a comparable strong influence on the rotarod performance (*p* < 0.04), as C57BL/6J, tg-aSYN-B6 and tg-aSYN-mtNOD mice, respectively, were able to improve their performance with advancing age (for further information of the statistical analyses, please refer to the text in the Supplementary Material).

#### Pole Test

To evaluate parameters of dementia and motoric alteration, the pole test was chosen. Here, the mice need good spatial orientation and high motoric skills to turn 180° and climb head downwards to the ground. The best of all five trials was taken for further evaluation. Data of mice, which slipped or felt off or performed only parts of the pole, were not used for analysis.

Here, again a linear mixed model was fitted to statistically analyse the collected data over the described periods in time, 25–33 weeks (mid-age) and 37–41 weeks (old age), respectively. Interestingly, analyses of mid-age points in time (25–33 weeks) displayed no significant changes in both determined parameters (climb latency and time-until-turn-latency) (*p* ≥ 0.06). Here, only a tendency of different climb performance was detectable, as tg-aSYN-mtNOD mice seemed to improve their performance as compared to tg-aSYN-B6 and C57BL/6J mice.

**Fig. 7:**
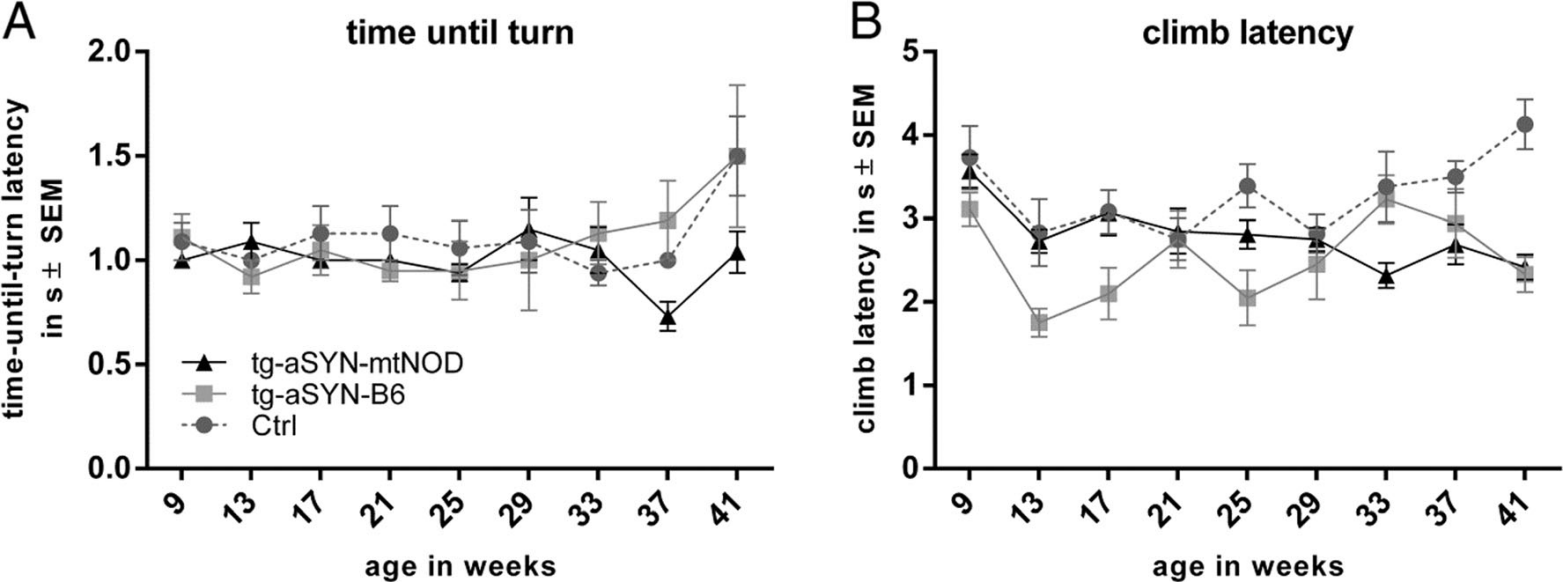
Changes in pole test performances of tg-aSYN-B6, tg-aSYN-mtNOD and C57BL/6J control mice. No significant alterations were detected in time-until-turn-latency (**a**) and climb latency (**b**) until old age. A better orientation performance (time-until-turn-latency) was found in tg-aSYN-mtNOD mice at older age, i.e. 37–41 weeks (**a**). At the same points in time, the climb latency surprisingly revealed reduced performance of control mice when compared to tg-aSYN-B6 mice (**b**). Data is presented as means ± SEM (*n* ≥ 5, male). (restricted maximum likelihood (REML) approach and additional two-sided *t* test with adjusted degrees of freedom)

However, we found mice at older age (37–41 weeks) to show more distinct differences, especially a significantly better orientation performances (time-until-turn-latency) in tg-aSYN-mtNOD mice (*p* = 0.0237) (Fig. 7a), and surprisingly a decreased climbing performance in C57BL/6J mice (*p* = 0.001) as compared tg-aSYN-B6 mice (Fig. 7b).

## Discussion

Mitochondrial dysfunction is one of the major hypotheses describing a causative role for PD. It is thought that during age, mutations in the mitochondrial genome (mtDNA) accumulate and lead to the excessive production of reactive oxygen species (ROS). ROS are implicated in a wide range of neurodegenerative diseases as they are thought to enhance the propensity of peptides to aggregate, e.g. β-amyloid, aSYN [17, 34, 35]. Evidence for the assumption of mutations in the mitochondrial genome being implicated in ageing were first brought using a knock-in mouse model with a mutated mtDNA-polymerase A [36]. A decreased lifespan and early onset of age-related phenotypes were described due to a deficiency in the proof-reading function of the protein [37]. Additionally, first hints were collected when complex I-deficiency in cells of the substantia nigra *pars compacta* was discovered in patients with Parkinson’s disease (reviewed in [5, 38]).

In the reported study, we analysed two genetically different tg-aSYN mouse strains expressing human aSYN with the ‘A30P’ mutation. The first one, the tg-aSYN-B6 strain, is a well-known and characterised model for cortical α-synucleinopathies [24]. The second strain, related from ‘non-obese diabetic’ (NOD) mice, has additional mitochondrial DNA polymorphisms (tg-aSYN-mtNOD). Mice with these variations were already investigated in the context of Alzheimer’s disease and formerly described to reveal a higher production of ATP [23]. As they are widely used in other fields of research, too, they are well characterised and have been sequenced previously [25]. Table 1 shows an overview of gene and amino acids changes of NOD/LtJ mice as compared to C57BL/6J mice.

**Table 1:** Mitochondria gene differences between NOD/LtJ and C57BL/6J mice (adopted from [25])

Gene	mtDNA position	C57BL/6J	NOD/LtJ	Amino acids
Cox3	9348 bp	G	A	Val > Ile
ND3	9461 bp	C	T	Met > Met
tRNA^Arg^	9821 bp	8A repeats	10A repeats	

We compared both transgenic mouse strains (tg-aSYN-B6 and tg-aSYN-mtNOD) with the C57BL/6J controls regarding aSYN load, immune response, neuron integrity and behavioural changes with the focus on differences due to mitochondrial polymorphisms. Although tg-aSYN-B6 mice tend to develop epileptic seizures with advancing age, we did not find phenotypic alterations as compared to control mice in the first place. The same holds true for tg-aSYN-mtNOD mice, which do not show any differences when compared to tg-aSYN-B6 and C57BL/6J mice.

Immunohistological analyses revealed a slightly more intensive immunolabelling with anti-human aSYN antibody (clone 5G4) in the brains of tg-aSYN-B6 than in tg-aSYN-mtNOD mice, indicating a higher aSYN burden. In contrast to findings of Kovacs et al. [39], who described no background or synaptic staining in brain sections of PD patients but strong labelling of intracellular and neuritic structures using this antibody and a slightly different tissue preparation, we observed a strong background staining in both mouse models. We found the synapses and somata in the brains neurons to be strongly labelled, probably because the artificial overexpression of human aSYN ‘A30P’ leads to the formation of mainly small oligomers enriched in synapses and somata rather than aggregates and fibrils which are the preferred targets of this antibody [39, 40]. In addition to this, it was described before that the overexpression of aSYN in mouse models does not lead to the formation of Lewy bodies-like in human brain tissue (reviewed in [41]). As expected, anatomical structures were strongly stained including those implicated in memory, behaviour and motoric skills like the hippocampus, amygdala, somatosensoric cortex and thalamus. Here, no differences in intensity could be determined between young and old tg-aSYN-B6 and tg-aSYN-mtNOD mice, respectively (not shown). Furthermore, only little differences between old tg-aSYN-B6 and tg-aSYN-mtNOD mice were detectable, whereby old tg-aSYN-B6 display a slightly more intense staining in the cortex, leading to the impression that the aSYN load is greater in tg-aSYN-B6 mice. Due to the expression pattern of the Thy1-promoter, several structures sparse any immunolabelling including substantia nigra and the olfactory bulb, both prominently affected in post mortem brain tissue of PD patients, but were described earlier to lack aSYN in transgenic PD mouse models [42]. C57BL/6J mice show no immunoreactivity at all. The strong, almost ubiquitous staining complicates a definite statement of differences in aSYN load and distribution between both transgenic mouse models.

To define differences in aSYN burden, we performed ELISA measurements. Surprisingly, we found the tg-aSYN-mtNOD mice to have significantly increased guanidine-soluble aSYN (higher molecular species) in the brain as compared to tg-aSYN-B6 mice. Unexpectedly, we did not observe any difference in the amount of carbonate-soluble (monomers and small oligomers) aSYN in the brain between tg-aSYN-B6 and tg-aSYN-mtNOD mice, neither at 50 days nor at 300 days of age. Noticeably, an increase in aSYN concentration (monomers/small oligomers and higher molecular species) with advancing age in the brains of both transgenic mouse models was missing, instead a tendency to decreased aSYN measured in the brain of 300-day-old mice compared to 50-day-old ones could be revealed. Together with the aforementioned missing increase in aSYN staining in young and old tg-aSYN and tg-aSYN-mtNOD mice, it became thinkable that mechanisms exist which remove aSYN continuously from the brain and lead to an equilibrium between production and removal, resulting in the observed steady state of aSYN burden in the brain of young and old tg-aSYN and tg-aSYN-mtNOD mice, respectively.

In this context, the combined finding of old tg-aSYN-mtNOD having an increased immune response (Iba1) as compared to controls and the neuronal integrity in aged tg-aSYN-mtNOD mice being rescued to levels of age-matched C57BL/6J mice is most interesting. Although the number of neurons is drastically reduced in all strains, indicating that the degeneration process is part of the normal ageing, the neuronal area of old tg-aSYN-B6 mice is reduced by further 35 % compared to age-matched control mice. A conceivable cause for the neuronal degradation is the impaired aSYN functionality and the resulting loss of function as modulator of synaptic activity. As aSYN binds to synaptic vesicles and serves as part of the SNARE-complex [43], it is most likely that alteration leads to neuronal degradation as common mechanism. Burré et al. [44] recently showed that the ‘A30P’ mutation is unique among the aSYN point mutations in impairing SNARE-complex formation.

Taken together, these findings emphasises the supposition of higher molecular forms of aSYN being less toxic than the smaller soluble moieties, and the aggregation to be a mechanism that mediates protection of neurons from further damage. This observation gets well along with proposals made by several other research groups and in the context of different neurodegenerative diseases [45–47].

These findings further support the theory of a neuroprotective effect emanating from microglia, a topic which has been controversially discussed (reviewed in [48]) especially in the context of mitochondrial influence in neurodegeneration [49].

To verify, if the described changes in tg-aSYN-mtNOD mice are not only histological phenomena but have indeed a beneficial impact on motor skills, behavioural tests were performed. Firstly, tg-aSYN-B6 mouse models display age-dependent movement abnormalities as previously described [50]. Freichel et al. [42] found old tg-aSYN-B6 mice to be significantly worse in the fear conditioning and active avoidance tests, in which the involvement of the amygdala and hippocampus is required. Interestingly, these mice show a worsening in the Rotarod performance test, but starting not earlier than 12 months of age. As we were mainly interested in earlier time points, we concentrated on the age of 50 to 300 days and could verify no differences between tg-aSYN-B6 and control mice as well. The cut-off time of 240 s in the Rotarod performance procedure provides some difficulties in the statistical analysis, but gave interesting results on its own. The rate of censoring, i.e. of reaching the highest possible time on the rod without falling off, differs tremendously between transgenic mice. As expected, tg-aSYN-mtNOD mice achieved significantly better results possibly due to their intact neuronal integrity.

Intriguingly, we found tg-aSYN-mtNOD mice to perform significantly better in the Rotarod performance test as compared to tg-aSYN-B6, with the most tremendous differences at mid-age points in time (25–33 weeks).

Furthermore, we propose that age plays a significant role in this process. Surprisingly, both mouse models, tg-aSYN-B6 and tg-aSYN-mtNOD, show a positive increase in their performance with advancing age, with the highest increase for tg-aSYN-mtNOD mice. These results further confirm the notion of tg-aSYN-mtNOD mice to have a great benefit from the effects elicited by their mitochondrial polymorphisms.

The pole test could reveal similar results. The tg-aSYN-B6 mice show a significantly decreased time-until-turn performance at old age (37–41 weeks) as compared to tg-aSYN-mtNOD mice. The latter show a stable level of performance. The time the mice needed to climb down the wooden pole to reach the floor varies strongly among the age groups. Statistical significance was only reached for the increased performance of C57BL/6J mice.

The Rotarod and pole test are methods to examine coordinated motor skills and are widely used to detect nigrostriatal damage and dopaminergic denervation [51–53]. The missing difference between our tg-aSYN-B6 mouse model and the C57BL/6J (Ctrl) mice is founded in the lack of SNpc pathology due to the expression pattern of the Thy1 promoter, described before [50, 54]. The tg-aSYN-B6 mouse was therefore found to be not suitable to model purely motor disease aspects of PD, but useful to reproduce extranigral facets of PD and additionally dementia with Lewy bodies [50, 54].

The strong difference between tg-aSYN-B6 and tg-aSYN-mtNOD mice in the rotarod performance but missing variance in the climb performance of the pole test can be explained by the less phenotypic alteration in the transgenic mice. This very sensitive pole test [51] is most likely not suitable for extranigral aspect determination and stronger incentives for the mice are needed for future analyses. However, the ability of spatial orientation, a non-motor task, as part of the Pole test was disturbed in old tg-aSYN-B6 and control C57BL/6J mice, and was increased in tg-aSYN-mtNOD mice. Thus, a strong positive effect on the motor performance and spatial orientation task was found in tg-aSYN-mtNOD mice, confirming the described results.

To evaluate the influence of mitochondrial polymorphisms, we investigated brain mitochondria isolated from mice, by means of high-resolution respirometry with substrate inhibitor titrations [55, 56]. To obtain a maximum of information, we developed a new set of four protocols on the basis of longstanding experiences and the discovery of new functional properties of brain mitochondria [26, 28, 33, 55, 57–59]. This allowed the precise determination of (i) maximal substrate specific rates of state 3 respiration, (ii) maximal rates limited by the capacity of the respiratory chain complexes, (iii) respiratory control ratios, (iv) substrate specific Ca^2+^ stimulation of state 3 respiration and (v) resting rates of respiration under three different conditions. Interestingly, we mainly detected differences in succinate-dependent parameters of the mitochondrial function in old animals: (i) the rate of state 3_suc_ respiration was about 20 % lower in 200-day-old control mice than in the presence of NOD mitochondria and lower in both groups of young mice (C57BL/6J and mtNOD mice, respectively). (ii) For the same reason, the ratio of complex I/complex II-limited respiration was increased by 15 % in C57BL/6J (control) mice as compared to old mtNOD mice. (iii) Moreover, the rates of state 4_complex II_ respiration were also lower in brain mitochondria from 200-day-old C57BL/6J (control) mice whereas the rates of state 4_complex I_ respiration were not changed in any of the groups. The repeated findings of diminished succinate respiration in C57BL/6J mice using four different protocols (and starting parameters) indicates that there are indeed metabolic conditions causing reduced succinate-dependent rates of respiration which do not occur in the mtNOD mice.

What could be the reason for the diminished fluxes through complex II?

It is known that the activity of SDH, which oxidises succinate-forming fumarate, is dependent on the concentration of oxaloacetate and malate, both inhibitors of SDH [60]. This mechanism can explain why the succinate respiration as measured with α-ketoglutarate/malate (protocol II) was at least 40 % lower than measured with glutamate/malate in protocol I (Fig. 2). It is probably caused by different concentrations of oxaloacetate in both kinds of incubation. The mitochondrial aspartate amino-transaminase (ASAT) catalysing the reaction (glutamate + oxaloacetate = α-ketoglutarate + aspartate) can work in two directions: either decreasing the oxaloactetate concentration if reacting with glutamate or forming oxaloacetate if reacting with α-ketoglutarate. Therefore, using the glutamate containing incubation protocol, the oxaloacetate concentration is lowest and not inhibiting SDH, whereas in the presence of α-ketoglutarate, oxaloacetate it is at maximum, partially inhibiting SDH. If this explanation is correct, the reduced rates of succinate respiration found for old C57BL/6J mice in the glutamate containing protocol I (Fig. 2e) cannot be caused by an increased oxaloacetate concentration. Therefore, we assume that this effect could be caused by a diminished activity of SDH or the electron-transferring flavoprotein-ubichinone oxidoreductase (ETF), possibly caused by increased oxidative stress. In the old mtNOD animals, the rate of non-phosphorylating respiration with succinate is higher than in the controls. This could cause a reduced ROS formation under conditions where the SDH or the electron-transferring flavoprotein-ubichinone oxidoreductase (ETF) is especially required. Although succinate is not an important substrate for feeding the brain’s mitochondria, succinate is an important metabolite of the citrate cycle, and any limitation in its oxidation capacity can limit the oxidation capacity of the citrate cycle and limit the supply of reducing equivalents to the respiratory chain.

Even if there is no explanation for this interesting phenomenon today, there is no doubt that these findings reveal additional insights into the network of mitochondria and neurodegenerative diseases.

Taken together, we can infer that altered complex II-respiration potentially accompanied by an increased ATP production caused by mitochondrial polymorphisms occurs together with and may be responsible for improvement of motoric function and neuron integrity in tg-aSYN-mtNOD mice over a specific period of time, indicating disturbed ATP-dependent mechanisms like peptide degradation/removal and impaired SNARE-complex formation in the first place and again emphasising the important role of mitochondria in neurodegenerative diseases. This is conceivable as the brain is the largest consumer of energy in the body and it maintains its metabolism at equilibrium states.

A disturbed balance between influx and efflux of various substances and metabolites between blood and brain as provided by the transport via ATP-binding cassette transporter (ABC transporter) is crucial for the development of pathological conditions (reviewed in [61]). For example, the known ABCB10 transporter was recently identified to contribute to the prevention of oxidative stress by the export of yet unknown peptides in the inner mitochondrial membrane (reviewed in [62]). A decreased activity might additionally lead to mitochondrial dysfunction. Evidence was furthermore given by several studies for ABC transporter activity to be decreased in neurodegenerative diseases [29, 63]. This decreased activity is thought to lead to diminished detoxification of disease-associated regions in the brain, e.g. substantia nigra in Parkinson’s disease, hippocampus/temporal cortex in Alzheimer’s disease and caudate nucleus in Huntington’s disease [64].

In conclusion, alterations in the mitochondrial genome may pose benefits for the physiological fitness of mice overexpressing aSYN as seen in the Rotarod performance test and for the neuronal integrity as evidenced by immunohistochemistry. Thus, pharmacological intervention in mitochondrial function should be further examined in order to develop new strategies for disease modifying therapies.

## Electronic supplementary material

Below is the link to the electronic supplementary material.

ESM 1 (DOCX 29 kb)

## Abbreviations

ADP: Adenosine diphosphate
ATP: Adenosine triphosphate
ATPase: ATP synthase
ASYN: Alpha-synuclein
DTT: Dithiothreitol
EGTA: Ethylene glycol tetraacetic acid
ETC: Electron transport chain
FCCP: Carbonyl cyanide-4-(trifluoromethoxy)-phenylhydrazone
MOPS: 3-(*N*-morpholino)-propanesulfonic acid
PD: Parkinson’s disease
ROS: Reactive oxygen species
SDH: Succinate dehydrogenase
TMPD: *N*,*N*,*N*′,*N*′-tetramethyl-*p*-phenylenediamine
