# Supplementary material for: Activation of Mitochondrial Complex II-Dependent Respiration Is Beneficial for α-Synucleinopathies
Source: Mol Neurobiol. 2015 Aug 29;53:4728–44. doi: 10.1007/s12035-015-9399-4 (PMC4965489; doi:10.1007/s12035-015-9399-4)
Supplement: Supplementary file 1 — (DOCX 29 kb) [file 12035_2015_9399_MOESM1_ESM.docx]

Supplementary Material

**Title: Activation of mitochondrial complex II-dependent respiration is beneficial for α-synucleinopathies (Fröhlich *et al.*)**

Supplementary Table 1:

Sequential order of substrate addition in the four experimental approaches (protocol I- IV)

| protocol I | protocol II | protocol III | protocol IV |
| --- | --- | --- | --- |
| BIM-1000 | BIM-1000 | BIM-0 | BIM-0 |
| 2 mM malate | 2 mM malate | 2 mM malate | 2 mM malate |
| 10mM glutamate | 10mM a-Ketoglutarate | 10mM pyruvate | 10mM glutamate |
| 0.006 mg/ml mitochondria | | | |
| 2mM ADP | 2mM ADP | 100µM ADP | 100µM ADP |
| 200µM Ca^2+^ | 200µM Ca^2+^ | 2mM ADP | 2mM ADP |
| 100µM Ca^2+^ | 100µM Ca^2+^ | 10mM glutamate | 10mM pyruvate |
| 10mM pyruvate | 10mM pyruvate | 10mM succinate | 10mM succinate |
| 1.5µM rotenone | 1.5µM rotenone | 4 x 25nM FCCP | 1.5µM rotenone |
| 10mM glycerol-3-phosphate | 10mM glycerol-3-phosphate |  | 125µM atractyloside |
| 10mM succinate | 10mM succinate |  |  |
| 5µM antimycin A | 5µM antimycin A |  |  |
| 2mM ascorbate  500µM TMPD | 2mM ascorbate  500µM TMPD |  |  |
| 5mM azid | 5mM azid |  |  |

Supplementary Table 2:

Metabolic states of mitochondria according to Chance and Williams 1955 (from Chance, B. and G.R. Williams, Respiratory enzymes in oxidative phosphorylation. III. The steady state. J Biol Chem, 1955. 217(1): p. 409-27.)

|  | State 1 | State 2 | State 3 | State 4 | State 5 |
| --- | --- | --- | --- | --- | --- |
| Characteristics | Aerobic | Aerobic | Aerobic | Aerobic | Anaerobic |
| ADP level | Low | High | High | Low | High |
| Substrate level | Low-endogenous | Approaching 0 | High | High | High |
| Respiration rate | Slow | Slow | Fast | Slow | 0 |
| Rate-limiting component | Phosphate acceptor | Substrate | Respiratory chain | Phosphate acceptor | Oxygen |

Supplementary method:

**Linear Mixed Model for Censored Data and R-package *lmec***

A linear model can be used to compare treatment groups or to reveal linear relationships in a data set. Examining the rotarod data two problems occurred and had to be solved.

The first one is that every animal had to pass the measurement on several days and three times per measurement day. So it is recommended to fit a linear mixed model with random effects to model these repeated measurements – to model the effect of each animal. A linear mixed model contains fixed effects and random-effects, which are assumed to be normally distributed, and an error for each observation. For detailed information see McCulloch, C.E. and Searle, S.R. (2004): Generalized, Linear, and Mixed Models. Wiley series in probability and statistics: Applied probability and statistics, Wiley, chapter 6.

We were interested in three general questions resulting in different linear mixed models:

1. The comparison of two groups ($i=1, 2$) regarding the mean $\mu_{i}$ was modelled by

$Y_{ijkl}=\mu_{i}+b_{ij}+\varepsilon_{ijkl}$ .

These two groups $i\in\{1, 2\}$ can be two treatment groups (tg-aSYN-B6 vs. tg-aSYN-mtNOD) or a treatment group and a control group (tg-aSYN-B6 vs. C57BL/6). $j\in\{1,\ldots,J_{i}\}$ is the animal in group $i$and $k\in K_{ij}\subseteq\{1,\ldots,9\}$ is the age group of the animal $j$ in treatment group $i$at the point of measurement. Note that the animals did not pass the measurements in all age groups. $l\in\{1, 2, 3\}$ is the number of repeated measurement of animal $j$ in group $i$ and age group $k$.

The random variable $b_{ij}$ simulates the random-effect for the observed animal $j$ in group $i$. The test problem is to check, if the means in both groups differ:

$H_{0}: \mu_{1}=\mu_{2}$ against $H_{1}: \mu_{1}\neq\mu_{2}$ .

1. The model to find out, if there is a significant linear influence of the age, is

$Y_{ijkl}=\mu_{i}+\beta\cdot\left( t_{k}-25 \right)+b_{ij}+\varepsilon_{ijkl}$ .

As stated above $i\in\{1, 2\}$ is the group, $j$ is the animal, $k$ is the age group and $l\in\{1, 2, 3\}$ is the number of repeated measurement. $\beta$ is the slope and $t_{k}$ is the age (in weeks) of the animals in age group $k$. The age was centred in the model. $b_{ij}$ represents the random-effect for the animal. The test problem is to check, if there is a significantly from 0 different slope regarding to the age of the animals,

$H_{0}: \beta=0$ against $H_{1}: \beta\neq0$ .

1. To estimate the mean in each group combined with the age group and to test, if the *time curves* in the groups differ significantly, the following model was considered:

$Y_{ijkl}=\mu_{ik}+b_{ij}+\varepsilon_{ijkl}$ .

Here two ($I=2$, e.g. tg-aSYN-B6 vs. tg-aSYN-mtNOD) or all three ($I=3$) groups $i\in\{1,\ldots,I\}$ were compared. Analogous to the notation above $j$ is the animal, $k$ is the age group and $l\in\{1, 2, 3\}$ is the number of repeated measurement. The test problem is to check, if the mean $\mu_{ik}$ in each group and age group differs from the mean $\mu_{k}$ in each age group over all groups:

$H_{0}: \mu_{ik}=\mu_{k}$ $\forall i\in\left\{ 1;\ldots,I \right\}, k\in\{1,\ldots,9\}$

against $H_{1}: \exists i\in\left\{ 1,\ldots,I \right\}, k\in\left\{ 1,\ldots,9 \right\}: \mu_{ik}\neq\mu_{k}$.

The second problem is that the rotarod data are (right-)censored. So the experiment was terminated at a fixed time limit if the event did not happen by this time bound.

That the observed data are distributed normally with censoring at 240 seconds was checked by a q-q plot. Hence it is justified to assume normally distributed, uncorrelated errors.

Because of the great number of censored data (about half of the data) censoring cannot be ignored and hence it was not possible to use standard R-packages to evaluate these linear mixed models. It was appropriate to use the package *lmec*, which allows censored normal responses. In 2009 this package was built by Vaida and Liu and is recommended for that purpose by Allignol, A. and Latouche, A. (2014): CRAN Task View: Survival Analysis http://cran.r-project.org/web/views/Survival.html, version: 2014-05-31, last view 2014-07-17.

But this package is only for left-censored data, whereas the observed values are right-censored. That was the reason for multiplying all data by $-1$ and transforming the *lmec*-results back into the given context.

So it was possible to calculate the estimates using R 3.0.2. To test the hypotheses the model and the model under the null hypothesis were evaluated and a likelihood ratio test was performed. In order to do this the package *lmec*, (Vaida, F. and Liu, L. (2012): Package ’lmec’. Linear Mixed-Effects Models with Censored Responses. http://cran.r-project.org/web/packages/lmec/lmec.pdf, last view 2014-07-16.), provides the (estimated) log-likelihood value $\text{\$}\text{loglik}$ for each model. Then the null hypothesis $H_{0}$ was rejected if

$$-2(\text{\$loglik}_{H_{0}}-\text{\$loglik}_{H_{1}})>\chi_{\nu, 1-\alpha}^{2}$$

with $\chi_{\nu, 1-\alpha}^{2}$ as the $(1-\alpha)$-quantile of the $\chi^{2}$-distribution with $\nu$ degrees of freedom. $\nu$ is the difference in the number of fixed parameters in the null hypothesis and alternative. In model a) and b) it is $\nu=1$ and in model c) it is $\nu=2\cdot9-9=9$ for two time curves and $\nu=3\cdot9-9=18$ for three time curves.

It must be mentioned that the estimated results vary a little bit. We suppose that it is because of the random effects, which have to be simulated and numerical errors.
